# Altered Spontaneous Brain Activity in Cortical and Subcortical Regions in Parkinson's Disease

**DOI:** 10.1155/2016/5246021

**Published:** 2016-06-19

**Authors:** Jie Xiang, Xiuqin Jia, Huizhuo Li, Jiawei Qin, Peipeng Liang, Kuncheng Li

**Affiliations:** ^1^College of Computer Science and Technology, Taiyuan University of Technology, Taiyuan 030024, China; ^2^Department of Radiology, Xuanwu Hospital, Capital Medical University, Beijing 100053, China; ^3^Beijing Key Lab of MRI and Brain Informatics, Beijing 100053, China

## Abstract

*Purpose*. The present study aimed to explore the changes of amplitude of low-frequency fluctuations (ALFF) at rest in patients with Parkinson's disease (PD).* Methods*. Twenty-four PD patients and 22 healthy age-matched controls participated in the study. ALFF was measured on the whole brain of all participants. A two-sample *t*-test was then performed to detect the group differences with age, gender, education level, head motion, and gray matter volume as covariates.* Results*. It was showed that PD patients had significantly decreased ALFF in the left thalamus/caudate and right insula/inferior prefrontal gyrus, whereas they had increased ALFF in the right medial prefrontal cortex (BA 8/6) and dorsolateral prefrontal cortex (BA 9/10).* Conclusions*. Our results indicated that significant alterations of ALFF in the subcortical regions and prefrontal cortex have been detected in PD patients, independent of age, gender, education, head motion, and structural atrophy. The current findings further provide insights into the biological mechanism of the disease.

## 1. Introduction

As a progressive, neurodegenerative disorder caused by degenerative changes in the dopaminergic neurons of the substantia nigra [1, 2], Parkinson's disease (PD) is clinically characterized by movement impairment, including tremor, rigidity, bradykinesia, and loss of postural reflexes [3]. However, the underlying neural underpins are still unclear for PD.

Resting-state low-frequency BOLD signal fluctuations are considered to be related to spontaneous neuronal activity [4]. As a measure of magnitude of spontaneous BOLD signal, amplitude of low-frequency fluctuations (ALFF) integrates the square root of the power spectrum in a low-frequency range (0.01~0.08 Hz) [5]. Evidences have demonstrated that ALFF is associated with field potential activity in local brain regions [6], and reliable correlation between ALFF and regional cerebral blood flow (CBF) is found in most of gray matter areas [7]. Additionally, the test-retest reliability of ALFF has been verified in healthy volunteers [8]. To date, ALFF has been widely used to study the baseline activity and to identify the sensitive marker of neurodegenerative disease [9, 10].

Recently, evidences have suggested the potential role of ALFF as a marker in Parkinson's disease (PD) [11–14]. All these studies reported the dysfunctional activity of corticostriatothalamic circuits in PD [15–17]. However, the altered ALFF in this motor circuitry is heterogeneous and conflicting. For example, in the thalamostriatal system, one study found decreased ALFF in the thalamus and increased ALFF in the caudate [11], while another study found decreased ALFF in the thalamus and caudate [12]. These ALFF changes in the motor circuits for PD patients required further examination.

Additionally, the inconsistent results also existed in the prefrontal regions. For example, an increased ALFF in the medial prefrontal cortex was detected in [12], whereas an opposite pattern of ALFF change in this region was observed in [13, 14]. Although the heterogeneity of PD patients may have an effect on the imaging results, it was argued that the confounding factors including head motion and structural atrophy mainly contribute to the incongruent results. It was found that all the previous studies did not control for the possible effects of these confounding factors. Together, the ALFF changes in PD are still required to be clarified. 

The aim of present study was to investigate the altered spontaneous neuronal activity as measured by ALFF in PD patients (in contrast to healthy controls) after controlling for the potential nuisance factors.

## 2. Materials and Methods

### 2.1. Subjects

Twenty-four right-handed PD patients (12 males, 62.7 ± 7.4 years), who were recruited from the outpatient neurology clinic of the Xuanwu Hospital, Capital Medical University, and 22 right-handed healthy controls (11 male, 65.6 ± 6.9 years) participated in the current study. This study was approved by the Medical Research Ethics Committee of Xuanwu Hospital. Written informed consent was obtained from all participants.

The clinical diagnosis of PD was confirmed according to the UK Parkinson's Disease Society Brain Bank criteria [18]. All subjects were off medication for imaging and neuropsychological testing. All participants underwent the Mini-Mental State Exam (MMSE [19]), the Montreal Cognitive Assessment (MoCA, [20]), and University of Pennsylvania Smell Identification Test (UPSIT, [21]) to measure general cognitive and olfactory abilities. Only PD patients with normal cognitive function as defined by a score on the Mini-Mental State Examination (MMSE) ≥ 24 were selected. Disease severity was recorded using Movement Disorder Society-Unified Parkinson's Disease Rating Scale (MDS-UPDRS) [22] and Hoehn and Yahr (H&Y) staging [23]. The demographic and clinical characteristics of the subjects are listed in Table 1. 

### 2.2. MRI Data Acquisition

MRI data were collected on a SIEMENS Trio 3-T scanner (Siemens, Erlangen, Germany). Foam padding and headphones were used to limit head motion and reduce scanner noise. The subjects were instructed to hold still, keep their eyes closed, and think nothing in particular. Functional images were collected axially by using an echo-planar imaging (EPI) sequence (TR = 2000 ms, TE = 40 ms, flip angle = 90°, field of view (FOV) = 240 mm × 240 mm, matrix = 64 × 64, thickness = 4 mm, and gap = 1 mm; 28 slices). The scan lasted for 478 s. 3D T1-weighted magnetization-prepared rapid gradient echo (MPRAGE) sagittal images (TR = 1900 ms, TE = 2.2 ms, TI = 900 ms, FA = 9°, matrix = 256 × 256, and thickness = 1.0 mm; 176 slices) were acquired.

### 2.3. Data Preprocessing

fMRI data were preprocessed using SPM8 software (Wellcome Department of Cognitive Neurology, London, UK; http://www.fil.ion.ucl.ac.uk/). The first 10 volumes of the functional images was discarded for the signal equilibrium. Images were corrected for differences in timing of slice acquisition, followed by rigid body motion correction to the median image. The high resolution structural image was coregistered with the mean image of the EPI series. The structural image was then normalized to the MNI template, and normalization parameters were applied to EPI images. After normalization, all volumes were resampled into 3 × 3 × 3 mm^3^ voxels. The participants we used had the maximum displacement less than 1.5 mm and the angular motion less than 1.5° for each axis. Spatial smoothing was conducted with an isotropic Gaussian kernel of 6 mm of full-width at half-maximum.

### 2.4. Structural Image Analysis

To control for the potential impact of structural atrophy on the functional activations, a voxel-based morphometry (VBM) analysis of structural MRI was performed. Individual structural images were coregistered to the mean functional images after motion correction using a linear transformation. The transformed structural images were then segmented into gray matter (GM), white matter (WM), and cerebrospinal fluid (CSF) by using a unified segmentation algorithm [24]. Individual GM maps were modulated to compensate for the effect of spatial normalization. After spatially smoothing with a Gaussian kernel of 8 mm FWHM, a two-sample *t*-test was performed on the smoothed GM intensity maps by taking age, gender, and education level as covariates. The statistical threshold was set at an uncorrected *P* < 0.001 with the cluster size > 5 voxels.

### 2.5. ALFF Analysis

ALFF analysis was performed by using REST software (http://restfmri.net/). After the linear trend was removed, the fMRI data were temporally band-pass-filtered (0.01 < *f* < 0.08 Hz) to reduce the very low-frequency drift and high-frequency respiratory and cardiac noise [4, 25]. The time series for each voxel was transformed to the frequency domain and then the power spectrum was obtained. The square root was calculated at each frequency of the power spectrum. This averaged square root was taken as ALFF [5]. For standardization, the ALFF of each voxel was further divided by the global mean of ALFF values within a brain mask, which was obtained from the intersection of the brains of all subjects' T1 images.

### 2.6. Statistical Analysis

Distributions of age, education level, MMSE, MoCA, and UPSIT between the two groups were compared by using two-sample *t*-tests, and chi-square test was applied to compare gender distributions. A two-sample *t*-test was performed to investigate the ALFF difference between the patients with PD and normal controls, using age, gender, education level, head motion, and gray matter volume as covariates. An uncorrected voxel-level intensity threshold of *P* < 0.01 with a minimum cluster size of 13 contiguous voxels was used to correct for multiple comparisons using AlphaSim (https://afni.nimh.nih.gov/pub/dist/doc/manual/AlphaSim.pdf). This yielded a corrected threshold of *P* < 0.05.

### 2.7. Correlations between ALFF and Neuropsychological Measures

Correlation analysis of ALFF values against the neuropsychological measures was performed for PD patients. Averaged ALFF values of each cluster with the significant group differences were firstly extracted. Then, partial correlation analysis was executed to examine the relationship between the ALFF values and neuropsychological indices (including disease duration, UPDRS, MMSE, MoCA, and UPSIT) in PD patients using SPSS software (SPSS, Inc., Chicago, IL).

## 3. Results

### 3.1. Demographic Characteristics

The demographic and clinical data are listed in Table 1. Only the UPSIT for patients with PD (20.7 ± 4.2) was significantly lower than that of normal controls (28.2 ± 7.3) (*P* = 0.008). No significant differences were found for the other characteristics/measures between the two groups. Additionally, there was no significant difference for head motions during scanning between the two groups based on a two-sample *t*-test (*P* = 0.751).

### 3.2. VBM Results

As compared to NC, significant structural atrophies were detected in the left superior frontal gyrus (BA 11/10), left paracentral lobule (BA 5), and left middle frontal gyrus (BA 6) in PD patients.

### 3.3. ALFF Results

**Figure 1:**
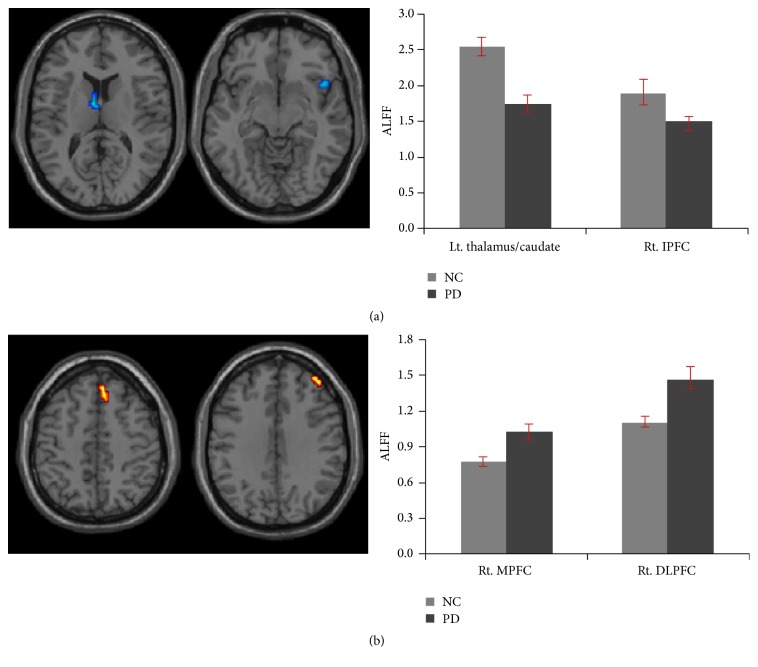
Regions of ALFF differences between PD patients and normal controls. (a) Decreased ALFF in PD patients and (b) increased ALFF in PD patients as compared to controls. MPFC: medial prefrontal cortex; DLPFC: dorsolateral prefrontal cortex.

Compared to normal controls, the patients with PD exhibited decreased ALFF in the subcortical regions including the left thalamus, caudate, and right insula/inferior prefrontal gyrus (Table 2, Figure 1(a)), whereas they exhibited the increased ALFF in the right medial prefrontal cortex (MPFC) (BA 8/6) and right dorsolateral prefrontal cortex (DLPFC) (BA9/10) (Table 2, Figure 1(b)).

No significant correlation was found between ALFF and the neuropsychological measures in regions with significant group differences.

## 4. Discussion

The current study examined the abnormal resting-state ALFF changes in PD patients after controlling for potential confounding factors including head motion and structural atrophy. Significant decreased ALFF in PD was identified in the striatothalamic circuitry and insula/inferior prefrontal gyrus, while increased ALFF was found in the MPFC and DLPFC. The reason we did not find the correlation between ALFF and the neuropsychological measures might be due to the long disease duration and the cortical reorganization for prolonged dopamine treatment [26].

### 4.1. Decreased ALFF in the Subcortical Regions

Dysfunction in the striatothalamic circuit was observed in the current study even after controlling for the possible nuisances including age, gender, education level, head motion, and gray matter loss. This is totally in line with previous studies of PD patients using ALFF [12, 13]. Additionally, the impairment of the striatothalamic circuit was also detected in PD patients using regional homogeneity (ReHo) [26, 27] and functional connectivity analysis [28–30]. Consistent with these previous studies, the decreased ALFF in the striatothalamic circuit (i.e., the motor pathway) may associate with the movement disorder in patients with PD.

There are evidences from neuroimaging studies linking the insula to nonmotor symptoms of PD [31, 32]. As the anterior insula is always involved in olfaction, thus, the decreased ALFF in the anterior insula in PD patients may explain the reduced olfactory function as reflected by the significant lower UPSIT in PD patients (as compared to controls). An alternative possibility is that the decreased ALFF in the anterior insula may relate to the cognitive impairment in PD patients, as this region is the key part of the salience network and plays an important role in the general cognitive functions [33, 34]. This explanation can be partially excluded, as PD patients in this study exhibited a relatively normal general cognitive function as measured by MMSE and MoCA.

### 4.2. Increased ALFF in the Prefrontal Cortex

In accordance with previous studies, the current study also observed the increased ALFF in the MPFC [13] and DLPFC [11] in PD patients. In addition, these cerebral regions were also identified to show increased activities in PD in contrast to NC using the ReHo method [35]. Given the important roles of the MPFC and DLPFC in many cognitive functions, such as strategy control [36] and conflict monitoring [37] for the MPFC, as well as executive control [38], working memory [39], planning [40], and reasoning [41, 42] for the DLPFC, it was argued that the increased ALFF in the prefrontal cortex may imply a compensatory mechanism in PD patients to maintain the relative normal cognitive functions. 

### 4.3. Limitations

In this study, TR of 2000 ms was used to cover the whole brain, the fluctuation effects of cardiac and respiratory function could be aliased into the low-frequency BOLD signal fluctuation [43]. The low-pass filtering could not completely remove the effects of such physiological noises. These aliasing effects may reduce the specificity of our findings or even confound the detected difference between the two groups. In future studies, these physiological effects could be included in the data analysis by recording the respiratory and cardiac cycles at the same time during MRI scanning. Additionally, the relative small sample size is another limitation of the present study.

## 5. Conclusion

In conclusion, the current study indicated the significant alterations of ALFF in the subcortical regions and prefrontal cortex in PD patients, which provided new evidences that the neuronal activity in the resting state has changed in PD. The current findings further provide insights into the biological mechanism of the disease.

## Figures and Tables

**Table 1:** Demographic and clinical characteristics of the PD patients and normal controls.

	PD	Control	*P* value
Age (years)	62.7 ± 7.4	65.6 ± 6.9	0.173
Gender (male/female)^†^	12/12	11/11	1.0
Education (years)	13.6 ± 3.1	12.9 ± 3.7	0.411
Duration of disease	7.0 ± 3.3	—	
H&Y	2.2 ± 0.9	—	
MDS-UPDRS Part III	22.0 ± 7.0	—	
MMSE	27.3 ± 2.1	28.6 ± 1.6	0.101
MoCA	25.9 ± 3.7	25.4 ± 2.5	0.702
UPSIT	20.7 ± 4.2	28.2 ± 7.3	<0.01

H & Y: Hoehn & Yahr staging; MDS-UPDRS: Movement Disorder Society-Unified Parkinson's Disease Rating Scale; MMSE: Mini-Mental State Examination; MoCA: Montreal Cognitive Assessment; UPSIT: University of Pennsylvania Smell Identification Test. *P* values were derived from Student's *t*-test comparing the two groups except for † that was derived using the chi-squared test.

**Table 2:** Brain regions exhibiting the significant altered ALFF between PD patients and normal controls, with age, gender, education level, head motion, and structural atrophy as covariates.

Regions	BA	Cluster	MNI			*T-*score
		Size	*x*	*y*	*z*	
*PD < NC*						
Rt. insula/inferior frontal gyrus	47	13	45	15	−9	3.78
Lt. thalamus		13	−6	−3	12	3.45
Lt. caudate			−6	6	6	2.60
*PD > NC*						
Rt. medial frontal gyrus	8	20	6	39	48	3.43
	6		6	30	42	3.28
Rt. middle frontal gyrus	9	14	42	45	33	3.30
Rt. superior frontal gyrus	10		45	54	21	3.26
